# The Olive Branch in American Politics

**Published:** 1888-06

**Authors:** 


### *THE OLIVE BRANCH IN MEDICAL POLITICS.*

In August, 1885, the Buffalo Medical and Surgical Association departed from the original purpose of its organization in devoting one or more of its meetings to the investigation of the accuracy of hospital statistics. This action was inspired by the publication at that time of the annual report of the Buffalo Hospital of the Sisters of Charity, which presented to the profession the record of its work for the preceding year, the success of which called forth the criticism of the friends of a rival hospital, and the Association was chosen as the most available avenue to give such investigation the widest publicity. At that meeting, the aforesaid report was referred to a special committee, which assumed the authority to interrogate the authorities of the hospital to search the records of the Board of Health, and to summon the medical and surgical staff as witnesses, in search of facts by which to justify their action. The committee presented to the Association a lengthy report, detailing the results of their fruitless search, and concluded by recommending for adoption a resolution directing "the attention of the members to the importance of accuracy and precision in the construction of hospital reports which are intended for publication."

As a consequence of this action of the Association, the members of the hospital staff, composed of many of the most influential members of the profession in the city, several of whom were ex-presidents of the Association, feeling the insult offered to the institution with which they were identified, and to themselves personally, have been conspicuous by their absence at the subsequent meetings of the Association, the attendance of which has dwindled down, until the Association, once the pride of the profession of Buffalo, has become a discredit and almost a disgrace to the profession of our city.

At the annual election in April last, an effort was made to harmonize the disaffected elements by the election of a member of the opposition to the position of vice-president, with a view to bring in the honorable professional gentlemen with whom he was identified. As a further inducement, at a meeting of the Association subsequently held, the following resolutions were adopted :

WHEREAS, The usefulness of this Association is, and has been, impaired by the non-attendance and co-operation of many of its members ; and,

*Whereas*, This withdrawal of support and attendance is caused by certain action taken by this Association in August, 1885, relative to the report of the Sisters of Charity Hospital, which action has been construed by said members as a reflection on their honesty and veracity ; therefore, in accordance with these facts, be it

*Resolved*, That it is the sense of this Association that no such construction was intended by the resolution adopted August, 1885, and that it is to be deplored that such misapprehension exists, and should have existed so long.

The points in the case may be briefly summarized as follows : The Association, in adopting the recommendation of the committee requiring "accuracy and precision in the construction of hospital reports," confirmed the doubt which interested parties had raised, and cast a reflection on the honor and veracity of the medical and surgical staff ; the Association has since suffered in usefulness and reputation in consequence of the non-attendance of these members of "questionable veracity ;" that after three years have elapsed, and time has worn away the bitterness of these aspersions, the Association condescends to construe what it formerly called a falsehood into a misapprehension, and to regret that such an impression should have existed so long.

We conclude by affirming that, among gentlemen, an apology should follow an insult, and that the honesty of purpose and abilities of well-bred professional men should not be wasted in an endeavor to resuscitate an association which has been so heavily freighted with "envy, hatred and malice" among its members, as to place it beyond the recognition of honorable men.

